# Narratives of young black men on barriers to health care and poor health care seeking behaviours at a university setting: a qualitative study

**DOI:** 10.1186/s12913-021-06470-9

**Published:** 2021-05-10

**Authors:** Sinakekelwe Khumalo, Musawenkosi Mabaso, Tawanda Makusha, Myra Taylor

**Affiliations:** 1 Discipline of Public Health, School of Nursing and Public Health Medicine, University of KwaZulu-Natal, Durban, South Africa; 2 Human and Social Capabilities Research Division, Human Sciences Research Council, Durban, South Africa; 3 DSI-NRF Centre of Excellence in Human Development, University of the Witwatersrand, Johannesburg, South Africa

**Keywords:** Access and utilization of health services, Black male students, university, South Africa

## Abstract

**Background:**

Institutions of higher learning provide education, training, independence and life-long skills for young people. However, for students to achieve their optimal growth and intellectual development they need to be healthy psychologically, mentally and physically. This can be achieved through the development of effective health programs for all university students. This qualitative study was designed to explore Black male students’ perspectives and experiences regarding the utilization of on-campus health services at the University of KwaZulu-Natal.

**Methods:**

The study population was selected using purposive sampling. Data were collected using four focus group discussions (FGDs) with 36 participants and three key informant interviews. Thematic analysis was conducted to identify the key patterns and themes that emerged from the data.

**Results:**

Emerging themes included poor knowledge and awareness, negative perceptions and attitudes, fear and lack of privacy, and negative experiences leading to poor access and utilization of campus health services. The findings suggested a need for more advocacy and awareness campaigns especially among first year students, campaigns for normalization of sexual health, addressing HIV stigma and discrimination, providing youth friendly services to improve students’ use of sexual health services, and ultimately, their overall health and well-being.

**Conclusions:**

The findings give valuable insights from male students on the barriers and potential solutions to campus health services and highlight where improvements can be directed to increase access and use of health services by the study population.

**Supplementary Information:**

The online version contains supplementary material available at 10.1186/s12913-021-06470-9.

## Introduction

In South Africa, over one million students are enrolled in universities while 700 000 students are registered at more than 50 technical vocational education training institutions and an additional of 90 000 students are registered at the many private higher education institutions around the country [1]. Institutions of higher learning are places where young people receive their education, training, and develop independence and life-long skills [2]. Promoting health is important for students’ academic performance [3]. As such, young people’s health must be given the adequate and appropriate attention it deserves [4]. When young people enter the university space, it is unclear if they have been adequately equipped for the many challenges that come with the dynamic environment of tertiary education such as sexual and reproductive health issues [5].

Campus health centres are in a unique position to positively influence students’ health behaviours and beliefs [6]. Many programs in institutions of higher learning have focused on providing knowledge, awareness and HIV related practices. However, such programs have been criticized for failing to adequately promote behavior change among university students [7, 8]. The university lifestyle is a shift towards greater freedom from the family life, and the social context of the university lifestyle exposes students to risky sexual behaviours which puts them at a higher risk of sexually transmitted infections (STIs) [9–11].

According to the 2012 South African National Household Survey report, young people age 15–24 were a high-risk group for HIV infection and in particular, young men were less likely to test for HIV or seek treatment [12]. Young people in institutions of higher learning are often considered as a high-risk population, because they are at an increased risk of acquiring STIs due to pressure to engage in high-risk behaviours such as excessive alcohol consumption, casual sex, and inconsistent condom use [13, 14]. Consequently, the promotion of health services utilisation for young people has gained the interest of public health over the years [15, 16] .

However, young people continue to face challenges and barriers when seeking health services. The inherent stigma embedded in young people’s sexuality and their poor access to sexual health services because of fear of being labelled as promiscuous has a negative bearing on young people’s utilisation of health services [17, 18]. The lack of information [19], stigma and discrimination [18], stereotypes [20] lack of services designed for male adolescents [21], the unfriendly attitudes of health care employees [7] are some of the barriers the policy and practice in health facilities [16, 22] .

Regarding young people at institutions of higher learning, many university and college campuses offer a range of sexual health services to prevent and treat STIs, decrease the risk of the health consequences of STIs, and promote positive sexual health practices among students [23]. If students encounter challenges and problems while accessing such services, such experiences are likely to influence their attitudes, perceptions and utilization of these services [24]. The campus/university space presents an important opportunity to understand students’ knowledge, risk perception and health-seeking behaviours [5, 25]. However, there remains a dearth of evidence-based solutions that aim to improve male student’s health seeking behaviour. Addressing access barriers would enable improved access to health care services and students’ overall health and well-being [24, 26]. This study explores the barriers faced by young men at the University of Kwa-Zulu Natal in accessing and utilizing on-campus health services. It also provides recommendations for enabling the development of interventions to address these barriers.

## Methods

### Study setting and approach

This is a qualitative study that was designed to gain an in-depth understanding of students’ perspectives and experiences of accessing and using health services. The study was conducted from September 2018 to November 2018 at the University of KwaZulu-Natal(UKZN). Participants were black male students who were selected using purposive sampling at UKZN Howard College Campus chosen because of its student composition which is rich in social, economic and cultural diversity. This campus is the largest and home to most of the faculties at the institution. Data were collected using focus group discussions (FGDs) and key informant interviews (KIIs) (see attached supplementary file).

Recruitment was done through the distribution of posters and flyers with the study description contact details of the researcher (SK). A snowball recruitment strategy was employed to recruit additional participants. The participants for the FGDs were recruited from the four Colleges at the Howard College Campus, namely, College of Agriculture, Engineering and Sciences, College of Health Sciences, College of Humanities and College of Law and Management studies. A total of four FGDs were conducted with a total of 36 male participants. Each FGD comprised of 8–10 participants and took about 90 min to complete. For the FGDs, the inclusion criteria were that all participants had to be Black (regardless of ethnicity), identify themselves as male, had to study at the university (both undergraduate and postgraduate students were included), and had to be between the ages of 18–30 years. The study first collected basic demographic information such as age and place of residence and then the focus groups were used to explore male students’ perspectives and experiences regarding the utilization of on-campus health services and probe barriers faced by young men at the University. Table 1 shows the demographic characteristics of the study sample.

**Table 1:** Demographical characteristics of the sample

Focus group discussion (FGD)	Level of study	Ages	Ethnicity	Sample size (N = 36)
**FGD 1**	First-year students	18–21	Zulu and Xhosa	10
**FGD 2**	s year students	19–23	Zulu and Xhosa	8
**FGD 3**	Third-year students	23–29	Zulu, Tswana, Sotho, and Xhosa	10
**FGD 4**	Post-graduate students (honours, masters and PhD)	24–30	Zulu, Xhosa, Venda and Sotho	8

KII were conducted with consenting participants working at the Campus HIV and AIDS Support Unit (CHASU). The unit is a UKZN HIV and AIDS prevention program that provides care, support and treatment services for the university’s thousands of students and employees (HIV and AIDS Support Unit, 2015). The program is located in all five campuses of the university and consists of HIV counsellors that are in the campus clinic and health promoters located at the Campus HIV and AIDS Support unit. The unit has several support structures for students such as peer education, women’s forum, men’s forum, positive living, abstinence forum and lesbian, gay, bisexual, trans, and/or intersex forum. One of the coordinators in the unit assisted with recruiting programme coordinators for KIIs. The KIIs included three coordinators (a health promoter, a men’s forum coordinator and a peer educator) from the unit. The key informants were able to reflect on their experiences of working in the unit as well as their interactions with students utilizing their services. All FGDs and KIIs were conducted in English, audio-recorded and transcribed.

### Data analysis

The FGDs and KIIs were audio recorded, and detailed notes were also taken. The audio recoded FGDs and KIIs were transcribed verbatim. Initially, the first author read all FGDs and the KIIs raw transcripts and developed an initial coding scheme, that was reviewed and refined by the second and fourth authors. The process of coding and analysis was guided by the thematic framework [27]. All audios, transcripts were analysed using the ATLAS.ti, which was used for line-by-line coding and grouping the initial codes into emerging themes.

## Results

The results of the study describe male students’ narratives of their perceptions and experiences of utilizing campus health services at the University of KwaZulu-Natal. The main themes that emerged from the FGDs and interviews are grouped under four categories, namely, lack of knowledge and awareness, negative perceptions and attitudes, fear and lack of privacy, and negative experiences leading to poor access and utilization of campus health services.

### Lack of knowledge and awareness

In the FGDs with the male students it became evident that most of them did not know CHASU and the services that were provided by the unit. This was particularly common among the first-year students who indicated they were not aware of any campus health services. Many students acknowledged difficulty in knowing where and how to seek help. Students lack awareness of health services on campus and this makes them vulnerable to distorted and misleading health information.

However, some first-year students did acknowledge that they had seen awareness campaigns held around campus which hugely focused on HIV testing and condom use, however, they reported that they did not attend such awareness campaigns. One of the first-year student participants reported the following:

“*Even when I see the campaigns on campus I hardly go to see what is happening, it is just not for me*” (FGD 1_1st year student).

One of the programme coordinators indicated that they make efforts to give information about their programs during the first year orientation. However, because some students do not come for orientation they miss out on the information they give out about their programs. Hence, first year student only gets to know about their programs late in the year or when they see them around campus. Nonetheless, the coordinator noted that they make effort to go around students’ residence to educate and provide information to students about their programs. This is highlighted in the following:

*“We normally have a presentation of our programs during orientation. However, the problem with orientation is that many students do not usually come to orientation, because they will be dealing with residential issues or funding, that’s why they are absent during orientation. It is only a few students that attend who orientation and they are the ones that are provided with information about our programs. We find that the is always a gap in lack of knowledge about our programs especially for the students who do not attend. So to bridge that gap we go to residence on-campus and off-campus to reach out to educate students of health-related issues”* CHASU, KII_2)

Another coordinator reported that in their efforts to involve students to be part of their programs, they invite them to be part of their peer education program. In our discussion, it came out that students’ interest in the program does not last.

*“The peer education program is one of the most important programs. Once a student becomes part of our peer education program we train them and invite them to workshops in efforts to educate to be good peer educators and peer mentors. It is an exciting program for many in the beginning you can see by their commitment, but somewhere, somehow I think that the love for peer education it does fade away among many of them. They end up not attending and some pull out because they get busy with their school work”.* CHASU, KII_1)

### Negative perceptions and attitudes

While some participants in the first year group reported their unwillingness to seek health care for fear of being seen walking out of the campus clinic by other students.

“*People will be looking at you, imagine coming out with pills while people are looking at you, they will see you with pills and think of HIV*” (FGD 1_1st year student).

Such perceptions affected their attitudes toward seeking health care. It also pointed out their lack of interest in engaging with programs advocated by the health unit. Participants who were in their second year of study to the postgraduate level stated that they knew about such services on campus. However, for some, even with their knowledge of the services offered by CHASU, they frowned upon these services because of the negative connotations attached to the unit by many students. Some participants indicated that they only came to know about CHASU and the on-campus clinic through their girlfriends. With the prevailing stigma associated with the campus health services, a student at postgraduate level indicated that he was more likely to consult with a friend or search online about safer sex practices and major illnesses than utilize the on-campus health services. The following excerpts further explain in detail:

*“I feel like they are going to tell me things that are known already. So, it’s like listening to the motivational speakers who will be telling me things I already know of. So, there is no use”.* (FGD 4_Post-graduate student)

Among the second-year students, there appeared to be competing views regarding the utilization of the campus health services. Some of the participants in the group agreed that they visited the unit to get information about condom use, sexually transmitted infections, HIV testing, book for medical circumcision and also attend debates that are held by the men’s forum in the unit. They reported that utilizing the campus health services benefited them a lot, as they were able to make informed decisions around safer sex practices and being responsible young men, through talks held by the men’s forum and during some of the activation campaigns which are sometimes held on campus.

*I go there to test for HIV testing, sexually transmitted infection and I know that a lot people go there for sexual related information…they are informative a lot* (FGD 2_2nd year student).

However, other young men in the same group indicated that they did not see the need for going to the unit to access health care service as they knew that they would be able to access sexual health information from the internet and their peers. Some stated that they self-administer the HIV test, hence they did not see a need to utilize the university health services.

“*I do not see the need for going there. I would not go there to get sexual advice. I can ask my friends or check for information on the internet. For HIV testing, I buy the kit and test myself privately at home*”. (FGD 2_2nd year student)

This suggested that a number of these young men preferred to consult with their friends with regards to health information. This was troubling as it indicated peer approval determined the health-seeking behaviour for these young men. Additionally, searching online could discourage seeking medical help, as it would prompt self-medication instead of seeking professional help. Self-testing for HIV alone without emotional support from a trained HIV councillor was troubling because it seemed to be driven by fear of consulting, stigma and discrimination.

Discussions with the coordinators revealed that a majority of male students held negative attitudes towards seeking help especially relating to their health.

*“Since I have worked here the male students don’t seek for help for any sickness… when we interview them or when we do counselling with them they will tell you that ‘let the body of a man heal itself without going to the clinic’”* (CHASU, KII_2).

This, according to the coordinators was a commonly held belief by most male students. They reported that on many occasions’ students and particularly male students would only utilise their services when they were critically ill. This resonated with accounts by some of the participants who reported that they were against seeking help when sick, because of cultural beliefs that a man needed to be strong at all times. The majority of the young men across all FGDs stated that they only went to seek help when “it got serious”. Some noted that they had sexually transmitted infections but it took them a long time to get it treated because they felt that it was not serious and that it would go away by itself.

*“For certain problems, I think the only time a man would want to consult another man or a doctor for that matter, it is when things are starting to get out of hand and I can’t handle it myself”* (FGD 3_3rd year student).

### Fear and lack of privacy

 When asked if they went for regular HIV testing, many participants indicated that they only did that if they had a one-night stand without using protection. A participant in the 3rd year focus group stated, *“My girlfriend is the one that tests, so if she is negative it means I am also negative”.* This emerged in all the FGDs, participants held the belief that if their girlfriends tested negative, it meant they were also negative.

This idea by the participants was confirmed by one of the key informants, who stated that male participants were scared of coming to their unit for HIV testing. He recounted that he had an encounter with a male student who came with his girlfriend but did not want to test, stating that.

“*I don’t have time and this is not for me and maybe my partner can test and I will hear from her how the test went” or “I am scared to test and will rather not know*” (CHASU, KII_1).

This was particularly troubling as some of these young men reported having multiple sexual partners and inconsistent condom use. The fear of testing highlighted the lack of information, knowledge and consideration for their partners. During FGDs some students perceived HIV as something that was far from them. The reason again was that most students linked health services to HIV. This was evident during discussions, as one participant narrated.

“*I am afraid of HIV I do not want to lie, I’m afraid of it but there are those people that have bad luck. Not because I am special, but sometimes luck really works [for me] and I’m not used to it”* referring to condoms (FGD 1_1st year students).

The location of the health services also emerged as a barrier for these young men to access the health services, as they reported that the location was not convenient enough for them. This confirms the idea that the location of health care services has an influence on whether the health care services are utilized.

*“The position of these places is a problem, people will be looking at you as you go in and come out, like when you go to the clinic and you are holding pills people are looking at you”* (FGD 2_2nd year student).

*“The position is extremely public, which is a problem because people know you on campus even if you don’t know the person but someone out there is looking at you. Saying so and so went to the clinic today So there is no privacy with regards to walking in and out” (FGD 4_Postgraduate-student*).

Participants stated that the clinic was previously positioned in a location that was not visible to people. Hence, they suggested that the clinic be re-positioned to its old location which was not visible to a lot of people. They were then able to walk in and out without being seen by other students. As it currently stands, the clinic is positioned in a busy, populated and noisy location on campus.

*The clinic used to be far away from where it is now, even though it is in a convenient position but everyone can see you walking in and out. It is next to the cafeteria, it is always noisy and busy around there.* (FGD 2_2nd year student).

### Negative experiences

Some participants described the negative experience with health care workers especially when they visited the campus clinic which then discouraged them from seeking health care services. When asked to elaborate on their negative experiences the common issue that emerged was lack of privacy and judgemental attitudes when they visited the campus clinic.

“*When you go there the service is bad they are rough even when you test and they will be asking you useless questions, like didn’t you know that having sex without a condom kills. It is embarrassing*” (FGD 4_Post-graduate student).

“*it is not like I am bad mouthing them or something, when you go there you will have to explain everything and sometimes you will have to speak to the nurse and there will be other people listening to what you saying… so I prefer visiting clinics back at home nurses there are nice*” (FGD 3_3rd year students).

Another challenge that emerged as a barrier to young men utilizing the health services was the operational hours of the health services. Participants reported that they finished their lectures late and at times they do not have time in between lectures to go to the clinic or to go to consult with coordinators at the campus health unit. Some reported that by the time they would be done with lectures they would either have to rush to take buses to the off-campus residence and by then most of the campus health service would be closed for the day.

*“There are days when I have to attend the whole day…sometimes I would see the campaigns on campus but because of lectures and sometimes submissions, it becomes hard for me to go”* (FGD 3_3rd year student).

Some students described the long waiting periods when visiting the clinic as a discouragement for them *“It’s not like 30 minutes, it is more than 30 minutes when you go there, you wait for so many hours”* (FGD 2_2nd year student).

Table 2 shows barriers and potential solutions suggested by male students based on their experiences on campus. These findings are meant to describe the barriers that hinder the utilization of campus health services in order to develop strategies that will enhance and promote the utilisation of campus health services. These findings will also facilitate the improvement of campus health services that are provided by UKZN.

**Table 2:** Barriers and potential solutions among male student at Howard Campus Colleges, university of KwaZulu-Natal, South Africa

Themes and barriers	Potential solutions
**Lack of knowledge and awareness about health services** • Students especially those in their first year were flooded with lots of information during orientation to remember information about health services	• Student representative council to continuously showcase these programs to students • Continued promotion advocacy and awareness about available services • Integration of health programs with the academic curriculum
**Negative perceptions and attitudes towards health services** • Mainly due to fear related to treatment for STI’s and testing for HIV • Limited sexual health information	• Campaigns to normalize sexual health and help-seeking on campus • Need strategies to sensitively engage male students • Change campus culture that promotes risk-taking behaviours
**Fear and lack of privacy** • Fear of being seen • Location and confidentiality • Current campus health services HIV/AIDS	• Campaigns to address HIV stigma and discrimination • Focus campus health services on sexual and reproductive health
**Negative experiences** • Judgmental or ‘having poor attitude • Odd working hours • Waiting time	• Providing youth friendly services • Improve service provider work environment • Review operational hours

## Discussion

The current study explored the barriers to health care and poor health-seeking behaviours by young Black men at the University of KwaZulu-Natal. The findings revealed that poor access and utilization of health services by male students was influenced by lack of knowledge and awareness, negative perceptions and attitudes, fear and lack of privacy, and negative experiences. The findings also revealed that the level of education at institutions of higher learning does not have an influence on the participants’ access and utilization of the health services on campus. These findings underpin the importance of understanding the challenges faced by male students in seeking health care. As such the study has implications for policy and health program planning for institutions of higher learning in the development and implementation of effective programs that appeal to the larger student population.

Like current findings others also found that reasons use and non-use of sexual health services among students were because they had limited knowledge and were unaware that services existed or did not know what was available [28, 29]. Lack of knowledge and awareness about available health services especially among first year students suggest a need for more health care provider interaction with the university community. Students need to be informed during orientation in their first year of the various services that are available at the university. However, another study with similar results found that “first year students felt overloaded with new information during their first-year orientation and found it difficult to remember information related to sexual health services throughout the year” [30]. This highlights the need for continued promotion, advocacy and awareness about available health services on campus.

However, even those who were knowledgeable about the campus health care service, deliberately did not utilise these health services which were freely provided for them. This was attributed to negative perceptions and attitudes towards available services on campus, which also reflects limited sexual health knowledge. This highlight a need for normalization of sexual health especially when it relates to HIV. Most importantly there is a need to change the campus culture that promotes risk-taking behaviours. This calls for radical health promotion and programmes which specifically target male students, to help improve help-seeking attitudes and the uptake of sexual health interventions [31] .

In addition, challenges related to fear and lack of privacy included the location of services on campus as it related to the confidentiality associated with testing for sexually transmitted infections and as HIV. This points to limited sexual health information. Their concerns with confidentiality were about the potential of being seen by friends and other members of the university community. However, Adams et al. argue that the locations of campus health services were positioned to make it easy for students to conveniently access and utilize their services [19]. Nevertheless, visibility of sexual health services should be coupled with normalizing sexual health on campus. The is also a need to strengthen and improve university communication and counselling strategies as it relates to HIV in general on campus.

In agreement with the current findings other studies showed that the youth in general reported avoiding services or that they had confidentiality concerns due to provider barriers [32]. Most of these studies described providers as judgmental’ or having a poor attitude [7]. Other frequently mentioned barriers to accessibility, included hours when services were offered coinciding with lectures, and the long waiting periods to receive services [33]. This highlights the need for continued training of service providers to provide improved youth friendly services that could provide students with capability, opportunity and motivation that influence campus health service use.

This study has certain limitations. Firstly, the study represents the voices of young male students from one race and coordinators from only one of the five university campuses. Secondly, the study focused on the Howard College Campus at the University of KwaZulu-Natal and the findings may not be generalised to other universities in in the he country. Thirdly, FGDs may have introduced a social desirability bias. Nevertheless, valuable insights have been gained from male students regarding the barriers and enablers of access and utilization of health services among study population.

## Conclusions

This study explored the perspectives and experiences of Black male student’s regarding the utilization of on-campus health services at the University of KwaZulu-Natal. The study confirmed that despite the availability of sexual health services at university health centres to promote sexual health, many male students delay or avoid seeking care. The main themes that emerged from the FGDs and interviews included poor knowledge and awareness, negative perceptions and attitudes, fear and lack of privacy, and negative experiences leading to poor access and utilization of campus health services. These findings highlight the need for continued promotion, advocacy and awareness about available services, campaigns to normalize sexual health and help-seeking on campus, as well as campaigns to address HIV stigma and discrimination, and the importance of improving service provider work environment. Identified barriers and potential solutions can be used to design targeted interventions to improve access and utilization of sexual health services on campus among the study population, and ultimately, to promote their overall health and well-being.

## Supplementary Information

Additional file 1.

## Data Availability

Data sharing is not applicable to this article as no datasets were generated or analysed during the current study.

## Abbreviations

AIDS: Acquired immunodeficiency syndrome
CHASU: Campus HIV and /AIDS Support Unit
FDGs: Focus group discussions
HIV: Human immunodeficiency virus
KIIs: Key informant interviews
STI: Sexually transmitted infections
UKZN: University of KwaZulu-Natal
