# Supplementary material for: Narratives of young black men on barriers to health care and poor health care seeking behaviours at a university setting: a qualitative study
Source: BMC Health Serv Res. 2021 May 10;21:445. doi: 10.1186/s12913-021-06470-9 (PMC8111892; doi:10.1186/s12913-021-06470-9)
Supplement: Supplementary file 1 — Additional file 1. [file 12913_2021_6470_MOESM1_ESM.docx]

**Narratives of young Black men on barriers to health care and poor health care seeking behaviours at a university setting: a qualitative study**

Sinakekelwe Khumalo ^1, 2,3^*, Musawenkosi Mabaso^2^, Tawanda Makusha^2,^ Myra Taylor^1^

^1^Discipline of Public Health, School of Nursing and Public Health Medicine, University of KwaZulu-Natal, Durban, South Africa

^2^Human and Social Capabilities Research Division, Human Sciences Research Council, Durban, South Africa

^3^ DSI-NRF Centre of Excellence in Human Development, University of the Witwatersrand, Johannesburg, South Africa

##
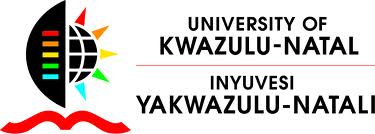


## FOCUS GROUP INTERVIEW GUIDE

## Manhood and Masculinity

1. In the community you are coming from what does it mean to be a man?

- What is a man in your own opinion? Probe: what makes a man to be a man in your opinion?
- How does society define being a man?
- Is it different from your family’s view of manhood?
- How is manhood achieved in your own opinion?
- What is your understanding of masculinity?
- What are the most desirable qualities or characteristics in your community regarding Masculinity or being masculine?
- What are the most undesirable qualities or characteristics in your community regarding Masculinity or being man?
- What are society’s expectations of a man?
- Are men generally able to live up to them? If yes explain and if no explain the challenges.
- In your community how are you perceived from other men who are not in university?

-probe for men employed: formally, informally and unemployed

## Manhood and sexual behaviour

In your community, what defines a man in terms of sexual behaviour? Probe for: Heterosexual, Homosexual behaviour.

- In your view what is the acceptable age for sexual initiation?
- What is important for you as a young men in terms of sexual relationships with your sexual partners? (love, looks or body)
- In the modern world, what is your opinion of men who sleep with other men? (Why do you say so?)
- Do men need to be in-love with someone before they have sex with them?
- In terms of number of sexual partners
- Who determines where, when and how to have sex and negotiating sex and safe sex? (in both heterosexual or homosexual)
- What does it man for men to have more than one sexual partner?

## Manhood and culture

1. Early childhood socialisation

- Who in your community determines the socialisation of boys and girls in their early year-probe, society and family (mother and father)?
- When growing up how were you socialised to be a man? Who did this socialisation?
- What is the role society, family (mother and father)?
- What are the prescribed roles for men and women?
- What happens if a man does not fit into the assigned roles? Probe: what happens if a man does not perform the assigned roles or is unable to perform the assigned roles.

1. Are there any traditional practices in your culture that teach and educate men about sex and sexual behaviours? If yes,

- What are these traditions/culture? –
- How do they shape and define manhood
- Who is responsible for teaching them?
- What impact do they have on you?
- Are these compulsory? If yes, what happens if one does not take part
- Do these traditions cater for different sexual orientation?
- How are they inclusive or discriminatory?

## University setting and sexual behaviour

1. Are men’s sexual behaviours different when they are in their communities and when they are in university? If yes explain how these are different (freedom, peer pressure)

## Sexual behaviour programming

1. Most students tend to experiment and have their first sexual encounters at university. May you tell me how the university setting has influenced your sexual behaviour?

- What are the types of male sexual behavioural programs that are in place in this university?
- Do men your age visit the University services for sexual health advice?
- How do men view these programs? Probe (Are they useful, how can they be improved)
- What would you recommend to deal with cultural norms associated with masculinity?
- What would you recommend to deal with cultural norms associated with sexuality?
- What are the barriers that hinder men from seeking health services?
- What can be done to encourage men to utilise health services?


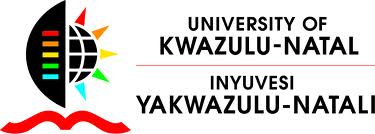


**KEY INFORMANTS INTERVIEW GUIDE**

1. In your area of work, oftentimes people speak about men’s health-seeking behaviours. Men usually do not go to clinics until they get extremely ill and they tend not to adhere to treatment if on treatment. Please tell us your views on views on men, masculinities and health-seeking behaviours among university male students.
2. Please tell us what you think causes young men at University to behave the way they do?
3. In your own view, how do cultural norms associated with masculinity influence on the sexual behaviour of male students?
4. Do you think young men at University present different forms of masculinity, sexuality and health-seeking behaviours?
5. What are some of the programs that are in place to promote healthy sexual practices and health-seeking behaviours among males in the university?
6. From your own experience, please tell me if these programs are being utilised by young men at the University? Please support your response.
7. How often do male students utilise some of these services?
8. Which age groups commonly use the services?
9. What are there barriers that hinder the utilisation of these programs?
10. In your opinion, what is it that the University leadership needs to do more than it is currently doing to promote healthy masculinities in terms of sexual behaviours among young men?
11. What are some of the programmes that are in place to promote healthy sexual practices among males in the university?
12. From your own experience to what extent are these programmes effective?
13. What are there barriers that hinder the effectiveness of these programmes in a university setting?
14. Is it different from a level of study?
15. What are some of the challenges that your encounter as coordinators in this unit? Probe: Human resources, funding, space, time
16. In your own view, how do cultural norms associated masculinity influence the sexual behaviour of male students?
17. What other additional measures would you recommend to deal with these challenges?
